# Online Navigation for Pre-Exposure Prophylaxis via PleasePrEPMe Chat for HIV Prevention: Protocol for a Development and Use Study

**DOI:** 10.2196/20187

**Published:** 2020-09-22

**Authors:** Shannon Weber, Laura Lazar, Alan McCord, Charlie Romero, Judy Tan

**Affiliations:** 1 University of California San Francisco San Francisco, CA United States; 2 PleasePrEPMe San Francisco, CA United States

**Keywords:** pre-exposure prophylaxis: PrEP, online chat, health systems navigation, HIV prevention, online navigation

## Abstract

**Background:**

Pre-exposure prophylaxis is an HIV medication taken by an individual who is HIV-negative to prevent infection before exposure to the virus. Numerous clinical studies in various communities have shown high rates of effectiveness when pre-exposure prophylaxis is taken as prescribed. Since FDA (US Food and Drug Administration) approval of the first product for pre-exposure prophylaxis in 2012, uptake has been lower than the estimated 1.1 million US adults who could benefit from its use, with an estimated 70,394 individuals on pre-exposure prophylaxis in 2017. Of these, only 11% were Black and 13% were Hispanic despite Black and Hispanic individuals comprising two-thirds of individuals who could benefit, highlighting racial and ethnic disparities in pre-exposure prophylaxis uptake. Patient navigators have been shown to be effective in improving the linkage and retention in care outcomes of people living with HIV across the HIV treatment cascade and can be used throughout the pre-exposure prophylaxis care continuum to assist decision making and connect potential users to pre-exposure prophylaxis services.

**Objective:**

PleasePrEPMe Chat was designed as a novel online strategy aimed at improving engagement in pre-exposure prophylaxis care services with pre-exposure prophylaxis–eligible populations in California via free HIV-prevention information and health care navigation services.

**Methods:**

Visitors connected with navigators via online bilingual (English, Spanish) chat. During the chat, navigators helped locate pre-exposure prophylaxis services through the PleasePrEPMe provider directory, provided links to HIV-prevention resources, and supported uninsured, insured, and undocumented visitors with benefits navigation. Data such as date, time, type of encounter, visitor type, key demographics, discussion topics, insurance, and other relevant information were collected via a chat log and through the HealthEngage chat platform.

**Results:**

From April 2017 to December 2019, PleasePrEPMe completed 2191 online chats. Mean interaction time was 16 minutes, with 68% of chats covering more than one topic. Conversation topics included health care navigation (1104/2191, 50.39%), provider identification (954/2191, 43.54%), pre-exposure prophylaxis information (773/2191, 35.28%), post-exposure prophylaxis information (318/2191, 14.91%), and the California Pre-Exposure Prophylaxis Assistance Program (232/2191, 10.59%). Referrals to pre-exposure prophylaxis– or non pre-exposure prophylaxis–related resources included directory updates, HIV testing and treatment, undetectable=untransmittable, reproductive health, sexually transmitted infections, and other prevention methods. A total of 368 chat visitors completed a voluntary satisfaction scale rating the quality and helpfulness of the service provided, producing a mean rating of 4.7 out of 5.

**Conclusions:**

Online chat is a method for reaching people not already engaged in HIV-prevention services, supporting HIV-prevention decision making, and linking people seeking information online with in-person services. Additional research to evaluate online sexual health information services and understand how social determinants of health influence online engagement is needed to better understand how to reach priority populations not well served by current HIV-prevention services.

**International Registered Report Identifier (IRRID):**

RR1-10.2196/20187

## Introduction

### Background

Pre-exposure prophylaxis is a regimen of HIV medications taken by an HIV-negative individual to prevent infection before an exposure to the virus. Numerous clinical studies in various communities have shown high rates of effectiveness when pre-exposure prophylaxis is taken as prescribed, as outlined in the US Public Health Service’s guidelines [1].

Since FDA (US Food and Drug Administration) approval of the first product for HIV pre-exposure prophylaxis in 2012, uptake has been lower than the Centers for Disease Control and Prevention’s (CDC) estimated 1.1 million US adults who could benefit from its use [2], with an estimated 70,394 individuals on pre-exposure prophylaxis in the fourth quarter of 2017 [3]. Black and Hispanic individuals comprise two-thirds of individuals that the CDC estimates could benefit from pre-exposure prophylaxis, yet 68% of pre-exposure prophylaxis users are white, while 11% are Black and 13% are Hispanic [2]. California lags behind the national average for decreases in new HIV diagnoses: from 2011-2016, the number of new HIV diagnoses fell 5.7% nationally, while the number in California dropped less than half of that—2.6% [4-6].

The pre-exposure prophylaxis care continuum is a framework by which to assess pre-exposure prophylaxis uptake and includes 3 phases: awareness (identify individuals at highest HIV risk, enhance self-perceived HIV risk awareness, raise pre-exposure prophylaxis awareness), uptake (facilitate pre-exposure prophylaxis access, link to pre-exposure prophylaxis care, prescribe pre-exposure prophylaxis, initiate pre-exposure prophylaxis), and adherence and retention (adhere to pre-exposure prophylaxis, retention in pre-exposure prophylaxis care) [7]. Although various pre-exposure prophylaxis care continua have been described and published [7-9], gaps exist due to the lack of scientific consensus on the best measures for pre-exposure prophylaxis program implementation.

Patient navigators have been shown to be effective in improving the linkage and retention in care outcomes of people living with HIV [10]. Similarly, patient navigators may provide support throughout the pre-exposure prophylaxis care continuum by assisting decision making and connecting potential pre-exposure prophylaxis users to pre-exposure prophylaxis services [11]. Navigation is generally defined as providing support for insurance, health care, and medication access and can range from a passive referral to active case management. HIV-prevention education and health benefits navigation may help ensure that potential pre-exposure prophylaxis users access adequate insurance coverage, state and local government pre-exposure prophylaxis services, and industry-sponsored co-pay and other medication assistance programs.

Internet usage is ubiquitous—across ages, races, and geographical regions [12]. Smartphone-based geospatial networking apps are a common method for finding sex partners among gay and bisexual men [13]. Rates of condomless anal sex and multiple sex partners are higher among people who connect with partners online [14], which highlights the potential impact of employing novel online strategies to improve engagement in pre-exposure prophylaxis care services with pre-exposure prophylaxis–eligible populations. Web-based interventions are increasingly being studied as methods for reaching individuals about sensitive topics and stigmatized issues such as HIV. For example, a web-based self-help intervention supplemented by brief chat counseling was shown to be an effective alternative to in-person counseling in supporting people with cannabis-use problems [15]. A systematic review [16] showed that electronic health interventions that address the prevention of HIV and other sexually transmitted infections (STIs) among men who have sex with men are impactful on changing short-term behavior.

### PleasePrEPMe Chat

The PleasePrEPMe website provides HIV-prevention information, training resources, and referrals, including a searchable, location-responsive pre-exposure prophylaxis provider directory [17] and tailored resource pages for potential pre-exposure prophylaxis users, frontline pre-exposure prophylaxis navigation staff, and medical providers. With this library of resources, PleasePrEPMe sought to expand web-based services to support people who seek HIV-prevention information and to link them to appropriate resources. PleasePrEPMe also features live chat services (PleasePrEPMe Chat) to reach potential pre-exposure prophylaxis users online in California and to facilitate their access to pre-exposure prophylaxis.

This paper describes the process of developing PleasePrEPMe Chat, an online chat service that helped potential users of pre-exposure prophylaxis understand their options and connect with pre-exposure prophylaxis–related care, as well as the quality assurance process and initial findings as of December 31, 2019. Specifically, this paper describes the multistep chat flow process by which PleasePrEPMe Chat engaged visitors online and the post-chat follow-up. 

## Methods

### Funding

PleasePrEPMe Chat was launched in April 2017, in collaboration with Project Inform and the Office of AIDS, California Department of Public Health, as a way for people to access bilingual confidential and free HIV-prevention information and health care navigation services online, removing the need to visit a clinic or provider for immediate health education and benefits navigation support. The California Department of Public Health provided funding to PleasePrEPMe. PleasePrEPMe subcontracted with Project Inform—a national community-based organization with 30 years of experience providing essential HIV treatment and prevention information (including a community helpline)—to develop this online model for providing health information and pre-exposure prophylaxis navigation services.

### Privacy Protection

PleasePrEPMe utilized various technologies and procedures to ensure the privacy of an individual’s personal health information, as defined in the Health Insurance Portability and Accountability Act (HIPAA) of 1996. (HIPAA establishes national standards to protect individuals’ personal health information and requires safeguards to protect the privacy of personal health information.) In support of these protections, PleasePrEPMe utilized the HIPAA-compliant, online chat management platform HealthEngage to administer the various aspects of on- and off-hours chat operations. The HealthEngage platform provided a user-friendly dashboard for PleasePrEPMe navigators to manage chats as it also records chat transcripts and collects internet-related data.

### Procedure

PleasePrEPMe Chat was staffed by one full-time bilingual (Spanish and English) navigator. Backup navigators were on call when chat visitors required one-on-one attention, chat volume was high, chat interactions transitioned to telephone calls, extensive research and follow-up was required, or the primary navigator was managing chats in multiple languages. Communications that support visitor navigation services among PleasePrEPMe staff, which include personal health information, took place through HIPAA-secure email or Zoom, a web-based video conferencing platform that complies with HIPAA standards. Real-time communications without personal health information took place in the messaging platform Slack.

Via online bilingual chat, navigators helped locate pre-exposure prophylaxis services through the PleasePrEPMe provider directory, provided links to HIV-prevention resources including educational material, and supported uninsured, insured, and undocumented visitors with benefits navigation. Open hours of operation for live chat were Monday-Friday, 9 AM to 5 PM (Pacific Time). Chats could be anonymous, although additional chats, email, text, and telephone follow-up were offered to visitors to augment the initial chat conversation or complete the navigation process.

During open hours, visitors with a California Internet Protocol (IP) address from their internet-access devices (computers, cell phones, other handhelds) who land on any webpage of the PleasePrEPMe website were offered *proactive chat*, whereby the chat box opened after 1 second on the web page offering to initiate chat. Automated messaging informed visitors that by engaging in chat they agreed to the website’s documented terms of use. Providing an email address was not required to chat, and chats could be completely anonymous. However, some passive data collection of IP addresses was undertaken by the HealthEngage platform. Visitors with IP addresses outside California and all visitors during offline hours could manually click on the chat button to complete a brief data collection form that generated an email message to PleasePrEPMe staff. The offline form included the user agreement for the terms of use. While PleasePrEPMe promoted chat as the primary method of online communication, staff could also be contacted for navigation services via the PleasePrEPMe website contact form, staff email, text, telephone, or social media.

### Chat Flow

**Figure 1:**
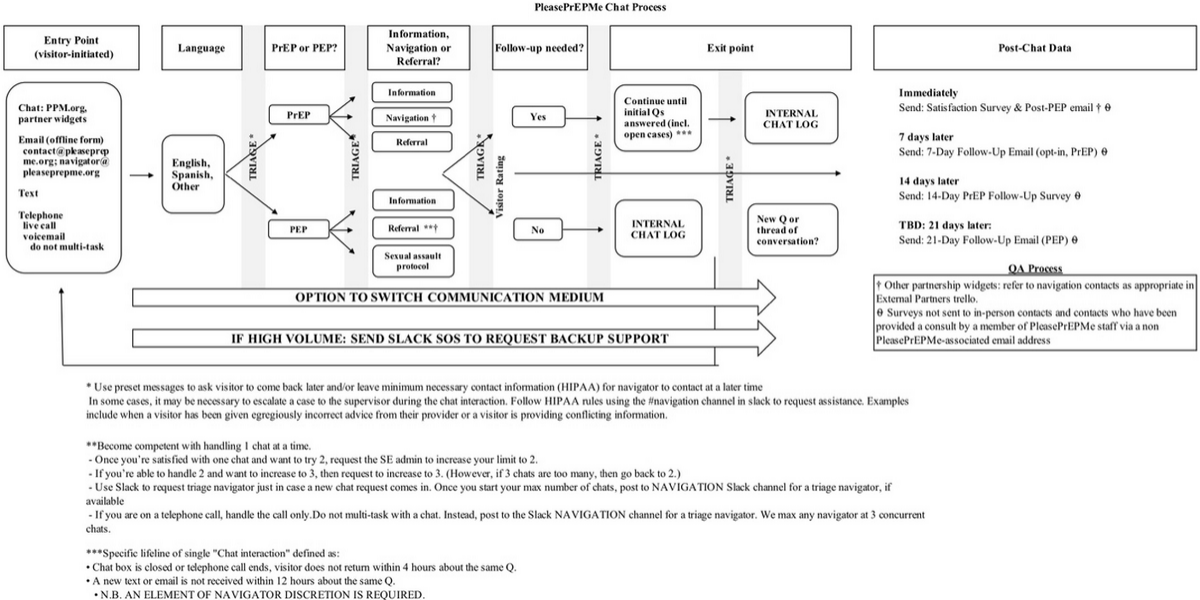
PleasePrEPMe Chat Process. PEP: post-exposure prophylaxis; PrEP: pre-exposure prophylaxis; Q: question; QA: quality assurance.

The chat flow chart represents a multistep process (Figure 1) by which visitors could engage with PleasePrEPMe online: service entry point, language screening, triaging post-exposure prophylaxis indication, core pre-exposure prophylaxis services, follow-up agreement, chat exit point, and post-chat follow-up.

### Entry Point

The primary entry point for a visitor into a PleasePrEPMe Chat encounter was through proactive chat or offline messages. Visitor emails received through the website’s contact form page, navigator email links, text message, telephone call, or voicemail could have been an entry point for a new encounter.

### Language Screening

Preferred language (English, Spanish) was assessed with each visitor through the HealthEngage platform, and Google Translate was utilized for other languages when needed.

### Post-exposure Prophylaxis Triage

PleasePrEPMe Chat encounters were initially triaged to determine if there was a recent HIV exposure within the previous 72 hours that warranted a conversation about post-exposure prophylaxis information and navigation rather than pre-exposure prophylaxis. (Post-exposure prophylaxis is a combination HIV regimen that is taken after a potential exposure to HIV, not before as with pre-exposure prophylaxis.)

### Core Services

Both pre-exposure prophylaxis and post-exposure prophylaxis encounters included services from 3 program categories: information provision (eg, HIV 101, pre-exposure prophylaxis 101, and other such basic information), navigation assistance (eg, health system information, insurance benefits, financial assistance programs), and referrals provision (eg, finding clinicians and colocated programs such as gender-affirming services). PleasePrEPMe chats often included support from more than one category. Additionally, a sexual assault protocol supported PleasePrEPMe staff with navigating applicable cases.

### Follow-up Agreement

Prior to closing the chat, the navigator assessed if the visitor desired follow-up information, such as receiving clinician contact information, detailed explanations of complicated insurance questions, referrals to assistance programs, or web links to additional content on discussed topics. Contact information—usually email—was collected for follow-up. The navigator would verify the information to be provided at follow-up.

### Chat Exit Point

At the conclusion of an encounter, the HealthEngage platform offered visitors a textbox with the option of providing feedback with a rating out of 5 stars. Once the chat was closed, PleasePrEPMe staff completed a PleasePrEPMe chat log (Textbox 1) using data collected through HealthEngage and chat content, such as date, time, type of encounter, visitor type, key demographic data, discussion topics, insurance, and other relevant information. Once the chat log was completed, if the visitor returned with an inquiry, it was treated as a new encounter, beginning at the entry point. Visitors could switch the PleasePrEPMe chat medium (ie, from chat to phone) and PleasePrEPMe staff could also offer this to a visitor, particularly in a post-exposure prophylaxis situation.

**PleasePrEPMe chat log.**

- Time stamp, date
- PleasePrEPMe staff member responding to initial request
- Medium initial request initiated via (chat, offline message, email message, telephone call, text, or other)
- Medium encounter continued via (chat, email, telephone call, text, or other), visitor contact information (email address or telephone number)
- Zip code
- City
- State
- Language (English, Spanish, other)
- Visitor type (pre-exposure prophylaxis or post-exposure prophylaxis user or potential user, non-clinical staff person, clinician)
- PleasePrEPMe chat topics covered (pre-exposure prophylaxis or post-exposure prophylaxis navigation, identified a specific medical provider/clinic, provider database entry or update, referral to non-navigation resources, treatment as prevention or undetectable equals untransmittable, reproductive health, HIV treatment 101, HIV testing/HIV 101, condoms and/or other prevention methods, other STIs, California’s Pre-Exposure Prophylaxis Assistance program, other)
- Initial question
- Insurance type (commercial, employer/private), Medi-Cal/Medicaid, Medicare, Veterans Administration, uninsured, not known, other
- Coding for level of PleasePrEPMe Chat (question was answered by PleasePrEPMe directory, question about sexual health or pre-exposure prophylaxis navigation, question required additional investigation and follow-up, question required post-exposure prophylaxis navigation or other emergency assistance, and non-navigation administrative question)

### Post-chat Follow-up

Once the PleasePrEPMe chat log was completed and if follow-up was needed, staff composed and delivered (eg, via email) the follow-up content as requested during the chat immediately for those seeking post-exposure prophylaxis and within 12 hours of the encounter for those seeking pre-exposure prophylaxis.

Depending upon the follow-up provided, one or two post-chat questionnaires (ie, Satisfaction Survey, 14-Day Pre-Exposure Prophylaxis Follow-up Survey) were sent using Google forms, and data automatically populated into a secure form on Google Drive. All questionnaires were available in both Spanish and English.

The first questionnaire (Satisfaction Survey; see Multimedia Appendix 1) was emailed to all visitors who provided an email address. The Satisfaction Survey assessed visitor experience with PleasePrEPMe chat service and referrals, identified how the visitor heard about PleasePrEPMe, and collected optional demographic information and feedback for improving services, including whether PleasePrEPMe helped them make decisions around their prevention needs. Visitors were given the option to provide their demographic information.

A second questionnaire, the 14-Day Pre-Exposure Prophylaxis Follow-up Survey (see Multimedia Appendix 2), was emailed to those who were provided referrals to clinicians. The 14-Day Pre-Exposure Prophylaxis Follow-up Survey was designed to assess the next steps in their access to pre-exposure prophylaxis care and prescription, including barriers they encountered, while providing another opportunity for PleasePrEPMe to continue supporting the visitor’s needs and receive feedback. PleasePrEPMe sent a 14-Day Pre-Exposure Prophylaxis Follow-up Survey to individuals who (1) provided an email address, (2) did not opt out of receiving future surveys, and (3) originally received a referral to a pre-exposure prophylaxis provider.

In January 2020, PleasePrEPMe emailed a retrospective questionnaire (see Multimedia Appendix 3) to the 1017 chat visitors who both chatted and provided email contact information between April 24, 2017 and December 31, 2019. The survey was designed to assess the utility of PleasePrEPMe’s services, gather feedback for service improvement, and understand other barriers to pre-exposure prophylaxis and post-exposure prophylaxis access. The survey was tailored to the experiences of potential pre-exposure prophylaxis users and frontline workers who assist individuals seeking pre-exposure prophylaxis with branching questions for each group (as self-identified) to answer. Individuals who did not remember their encounter with PleasePrEPMe were not eligible to respond. All eligible participants were offered the opportunity to opt into a drawing for a US $100 Visa gift card.

### Quality Assurance

Quality assurance protocols ensured the provision of accurate information and high levels of customer service throughout encounters with visitors. PleasePrEPMe employed several methods of quality assurance. First, at the close of a chat encounter, HealthEngage offered visitors a 5-star rating system with a textbox to provide optional feedback on their encounter. For any negative feedback received, staff reached out to those with an email address to provide additional support. Second, internal quality assurance reviews were performed on every encounter for new navigators and on a randomly selected number of encounters for experienced navigators. Internal quality assurance identified gaps in encounter consultations, resources or support for visitors, and areas for improvement and growth. Third, monthly internal quality assurance reviews were completed on a selected number of chats. The primary navigator assigned 3 to 5 transcripts and any follow-up emails to each team member for review. Team members utilized an Internal Quality Assurance Review Survey to review encounters (see Multimedia Appendix 4), such as providing suggestions for improvement (briefer responses, missed opportunities, use of transitional wording, more clarity, etc). Results of the monthly quality assurance review were discussed in team meetings, including recommendations for updates to protocols or content. Furthermore, PleasePrEPMe’s Director of Content and Quality provided quarterly reviews of the internal quality assurance forms, any negative feedback from surveys, and any challenging PleasePrEPMe chats flagged by staff. A report and discussion from this quarterly review was discussed in scheduled staff meetings. The chat process and post-chat follow-up questionnaires were approved by the University of California, San Francisco Human Research Protection Program Institutional Review Board.

## Results

From April 24, 2017 to December 31, 2019, PleasePrEPMe received 2991 chats; 755 were unrelated or incomplete chats, where incomplete information was received to allow for service provision, including truncated chats where PleasePrEPMe could not share information; consumer requests for information outside our areas of expertise; offline messages without enough data to reply; and system tests, and 45 chats were duplicate chats (for example, visitor left an offline message but also completed an online chat soon afterwards). We analyzed the 2191 complete chats. Chat user information is shown in Table 1.

**Table 1:** PleasePrEPMe Chat user information.

Category and type					Total, n (%)
**All users**					2991 (100)
	Unrelated or incomplete^a^				755 (25.24)
	Duplicate^a^				45 (1.50)
	**Complete**				2191 (73.25)
		**Service type (n=2191)**			
			Online live chat	1465 (66.86)	
			Offline chat form	309 (14.10)	
			Email	283 (12.92)	
			Telephone call or text	70 (3.19)	
			Other (social media, in person, etc)	64 (2.92)	
		**User location (n=2191)**			
			California	1674 (76.40)	
			Other states	422 (19.26)	
			Outside the United States	59 (2.69)	
			Unable to collect location data	36 (1.64)	
		**Language (n=2191)**			
			English	1960 (89.46)	
			Spanish	224 (10.22)	
			English and Spanish	5 (0.23)	
			Dutch (via Google Translate)	1 (0.05)	
			Chinese (via Google Translate)	1 (0.05)	
		**Visitor type (n=2191)**			
			Pre-exposure prophylaxis/post-exposure prophylaxis consumer or potential consumer	1585 (72.34)	
			Nonclinical staff person or frontline pre-exposure prophylaxis navigation worker	510 (23.28)	
			Clinical provider	80 (3.65)	
			Unknown	16 (0.73)	
		**Insurance (n=2191)**			
			Commercial insurance	447 (20.40)	
			Uninsured	302 (13.78)	
			Medicare/Medicaid	172 (7.85)	
			Other (Veterans Administration/TRICARE or student health insurance)	10 (0.46)	
			Multiple	9 (0.41)	
			Unknown	1251 (57.10)	

^a^Not included in subsequent calculations.

Online chats lasted a mean of 16 minutes (ranging from 8 seconds to 93 minutes), and topics varied across most chats while often covering more than one topic within chats (68%). PleasePrEPMe utilized an internal list of topic areas to categorize conversations. Topline topics included health care navigation (1104/2191, 50.39%), provider identification (954/2191, 43.54%), pre-exposure prophylaxis information (773/2191, 35.28%), post-exposure prophylaxis information (318/2191, 14.51%), and the California Pre-Exposure Prophylaxis Assistance Program (232/2191, 10.59%). Referrals to pre-exposure prophylaxis– or non pre-exposure prophylaxis–related resources included directory updates, HIV testing and treatment, undetectable=untransmittable, reproductive health, sexually transmitted infections, and other prevention methods. Chat content data are shown in Table 2.

**Table 2:** PleasePrEPMe Chat topic information.

Topic and description			Total^a^, n (%)
All chats			2191 (100)
Health care navigation and coverage options			1104 (50.39)
Identifying specific providers in the directory			954 (43.54)
Pre-exposure prophylaxis information, safety, effectiveness, side effects, dosing, adherence			773 (35.28)
Post-exposure prophylaxis information, safety, effectiveness, side effects, dosing, adherence			318 (14.51)
California Pre-Exposure Prophylaxis Assistance Program			232 (10.59)
**Referrals to pre-exposure prophylaxis– or non pre-exposure prophylaxis–related resources**			
	Directory updates	175 (7.99)	
	HIV testing or treatment/HIV 101	165 (7.53)	
	Undetectable=untransmittable	62 (2.83)	
	Reproductive health	44 (2.01)	
	Other STIs	34 (1.55)	
	Other prevention	26 (1.19)	
Not recorded			25 (1.14)

^a^Because a chat may include multiple topics, percentage will not equal 100.

### Quality Assurance and Improvement Measures

At the conclusion of a chat, within the chat platform visitors were offered an opportunity to rate the quality of the service on a scale ranging from 1 (not helpful at all) to 5 (very helpful) and to provide a comment. Of those offered the scale, 368 chat visitors participated, producing a mean rating of 4.7 out of 5.

Of 871 people who were sent the Satisfaction Survey, 85 (9.8%) responded. Of these, 75 (88.2%) responded “agree” or “strongly agree” to the statement, “PleasePrEPMe met my needs.” On the item, “How likely are you to refer a friend/colleague to PleasePrEPMe.org?” 75 (88.2%) responded “very likely” or “likely.” On the item, “PleasePrEPMe helped me make decisions about my HIV prevention, or helped me to help others around their HIV-prevention needs,” 67 people (78.8%) responded “agree” or “strongly agree.”

Of 515 people who were sent the 14-Day Pre-Exposure Prophylaxis Follow-up Survey, 25 (4.9%) responded. Of these, 19 (76%) had contacted a clinician since connecting with PleasePrEPMe, 10 (40%) had obtained a pre-exposure prophylaxis prescription, 7 (28%) were currently on pre-exposure prophylaxis, and all 7 were very satisfied with their decision to start. Reasons for not starting pre-exposure prophylaxis varied, with the reasons stated most frequently (4 of 14 given) being insurance and cost issues, provider delays such as long waitlists, not hearing back from clinics, or unsupportive providers.

Of the 1017 individuals who were sent a retrospective questionnaire, 74 completed questionnaires were received; 63/74 (85.1%) recalled their encounter—37 (59%) chatted on behalf of themselves, 25 (40%) on behalf of a client or for some other reason, and 1 (1.6%) did not remember. From the group of 37, 17 started pre-exposure prophylaxis or post-exposure prophylaxis after chatting, and PleasePrEPMe was able to help 14 of these respondents with getting or staying on pre-exposure prophylaxis or post-exposure prophylaxis. From the group of 25, PleasePrEPMe was able to help the client in 16 cases, 23 respondents were satisfied or very satisfied with PleasePrEPMe's knowledge level, and 21 were satisfied or very satisfied with PleasePrEPMe’s referral to resources.

## Discussion

Pre-exposure prophylaxis is a highly effective option to prevent HIV infection for those who are vulnerable to the virus and are aware of this relatively new development in biomedical prevention [1]. Employing patient navigators within medical and nonmedical services improves linkage to and retention in HIV care for people living with HIV [10]. Web-based interventions that aim to improve health outcomes around sensitive and stigmatizing issues such as sexual health and HIV are also increasingly being utilized [16,17]. PleasePrEPMe implemented a bilingual online program for people to access confidential, sex-positive, free HIV-prevention information and health care navigation services, with the goal to decrease the rate of new HIV infections [6,7]. PleasePrEPMe developed a chat program by incorporating several key approaches: offering a vetted search engine of pre-exposure prophylaxis providers, creating and providing data-driven content, utilizing available online technologies, adapting HIPAA-compliant standards to ensure privacy, and employing quality assurance methods to maintain and improve service provision.

Between April 2017 and December 2019, the PleasePrEPMe online chat navigation program (augmented by email, phone, and text) supported nearly 2200 individuals with sexual health questions. Potential consumers, frontline staff, and medical providers initiated a chat from various devices (laptop, cell phone, handheld) through a pop-up form on PleasePrEPMe’s and partners’ websites. PleasePrEPMe’s bilingual navigator tailored responses to questions and issues posed, and maintained chats until visitors had their needs addressed.

Online chat topics varied, with the topics most frequently discussed being health care navigation, medical provider identification, pre-exposure prophylaxis and post-exposure prophylaxis information, and patient financial support resources such as the California Pre-Exposure Prophylaxis Assistance Program. Topics also included HIV testing and treatment, undetectable=untransmittable, reproductive health, sexually transmitted infections, and other prevention methods.

Nearly half of the visitors received referrals. Satisfaction with the information and referrals provided by PleasePrEPMe was high. The majority of users believed they were helped with making decisions and that PleasePrEPMe met their needs. PleasePrEPMe was rated as highly helpful.

The nature of pre-exposure prophylaxis access challenges seen in California may be different and not generalizable to states without Medicaid expansion or where the overall culture may be more politically conservative. The issues and solutions around health care navigation that PleasePrEPMe encountered while assisting chat visitors from California may differ from those in other states. Additional research is needed to evaluate how novel services such as PleasePrEPMe are utilized by people who seek sexual health information and services online. Research can also help understand how social determinants of health [18] may or may not influence a person to engage in these services, and how to maximally leverage online marketing and outreach to reach priority populations currently underserved by traditional programs.

Online chat reaches people who were not already engaged in or need support with accessing HIV-prevention services. Chat also supported visitor education and decision making, linking individuals with appropriate in-person services. PleasePrEPMe aimed to reach people seeking to address their sexual health needs online, to support them with decision making, and to link them with in-person and online providers as needed. This approach could ease the need to visit a clinic or provider for health education and benefits navigation that can be handled online. It also helps to resolve challenges that some people encounter around pre-exposure prophylaxis access, including insurance and payment issues, confidentiality, and stigma.

HIV-prevention conversations must necessarily include pre-exposure prophylaxis and post-exposure prophylaxis navigation to holistically serve the needs of visitors at all stages in the pre-exposure prophylaxis care continuum.

## Appendix

Multimedia Appendix 1 PleasePrEPMe satisfaction survey.

Multimedia Appendix 2 PleasePrEPMe 14-day PrEP follow-up survey.

Multimedia Appendix 3 PleasePrEPMe retrospective survey.

Multimedia Appendix 4 PleasePrEPMe internal quality assurance review survey.

## Abbreviations

AIDS: acquired immunodeficiency syndrome
CDC: Centers for Disease Control and Prevention
HIPAA: Health Insurance Portability and Accountability Act
HIV: human immunodeficiency virus
IP: internet protocol
STI: sexually transmitted infection
