# Supplementary material for: Online Navigation for Pre-Exposure Prophylaxis via PleasePrEPMe Chat for HIV Prevention: Protocol for a Development and Use Study
Source: JMIR Res Protoc. 2020 Sep 22;9(9):e20187. doi: 10.2196/20187 (PMC7539157; doi:10.2196/20187)
Supplement: Multimedia Appendix 1 [file resprot_v9i9e20187_app1.docx]

|  | 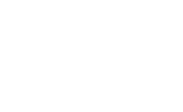 |
| --- | --- |
| **Survey 1:**  **Satisfaction Survey** |  |

**Thank you for contacting PleasePrEPMe.org!
We value your opinion. Please complete this survey to help us improve our services.**

**Whether or not you complete the survey and regardless of your answers, you are welcome to utilize PleasePrEPMe.org's services.**

*** Required**

1. Email address *

____________________________

1. PleasePrEPMe met my needs.*

*Mark only one:*

( ) ( ) ( ) ( ) ( )

Strongly disagree Strongly agree

1. Comments (optional)

____________________________

1. How did you hear about PleasePrEPMe?*

*Select all that apply*

Medical provider (nurse/doctor/other clinic worker including front desk staff and navigators)

Web search through google/bing etc.

Online ad through google/bing etc.

Social media (facebook/twitter/instagram etc.)

Dating and sex apps (Grindr/Scruff etc.)

Friend/family member

Other: ____________________________

1. PleasePrEPMe helped me make decisions about my HIV prevention, or helped me to help others around their HIV prevention needs.*

*Mark only one:*

( ) ( ) ( ) ( ) ( )

Strongly disagree Strongly agree

1. Comments (optional)

____________________________

1. How likely are you to refer a friend/colleague to PleasePrEPMe.org?*

*Mark only one:*

( ) ( ) ( ) ( ) ( )

Very unlikely Very likely

1. Comments (optional)

____________________________

The following helps us better understand PleasePrEPMe consumers.

1. What is your race / ethnicity?

*Select all that apply*

Asian/Pacific Islander

African American/Black

Latino/Hispanic

American Indian or Alaskan Native

White

Other: ____________________________

1. How old are you?

____________________________

1. What is your current gender identity?

*Mark only one:*

( ) Male

( ) Female

( ) Trans male/Trans man

( ) Trans female/Trans woman

( ) Genderqueer

( ) Decline to state

( ) Other: ________________________

1. What sex were you assigned at birth?

*Mark only one:*

( ) Male

( ) Female

( ) Decline to state

1. Do you have other feedback for us? Would you like to share more information about your experience?

____________________________

1. If you don't want to receive any communication from us again, please click here:

*Check all that apply.*

Please do not contact me

Thank you for telling us about your experience with PleasePrEPMe.org. We value your opinion.
