# Supplementary material for: Online Navigation for Pre-Exposure Prophylaxis via PleasePrEPMe Chat for HIV Prevention: Protocol for a Development and Use Study
Source: JMIR Res Protoc. 2020 Sep 22;9(9):e20187. doi: 10.2196/20187 (PMC7539157; doi:10.2196/20187)
Supplement: Multimedia Appendix 2 [file resprot_v9i9e20187_app2.docx]

|  | 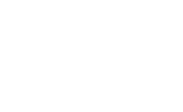 |
| --- | --- |
| **Survey 2:**  **14-Day PrEP Follow-Up Survey** |  |

**About two weeks ago, you contacted PleasePrEPMe.org. We are reaching out now with this follow-up survey because we provided you a referral to a PrEP provider(s).**

*** Required**

1. Email address *

____________________________

1. Have you contacted a clinician since connecting with PleasePrEPMe.org?

*Mark only one:*

( ) Yes

( ) No

1. How satisfied were you with the referral(s) to a PrEP provider?

*Mark only one:*

( ) ( ) ( ) ( ) ( )

Very dissatisfied Very satisfied

1. Please include any additional feedback here (optional):

____________________________

1. Did you have a medical visit with a PrEP provider?

*Mark only one:*

( ) Yes

( ) No

1. Did you get a PrEP prescription?

*Mark only one:*

( ) Yes - *Skip to question 8*

( ) No - *Skip to question 7*

PrEP was not prescribed

1. We understand that things get in the way of getting PrEP, or that people change their minds. Help us better understand your experience. Details are welcomed!

____________________________

PrEP was prescribed

1. Are you taking PrEP now?

*Mark only one:*

( ) Yes

( ) No

1. How satisfied are you with your decision to start PrEP?

*Mark only one:*

( ) ( ) ( ) ( ) ( )

Very dissatisfied Very satisfied

1. Please include any additional feedback here (optional)

____________________________

1. What challenges, if any, have you experienced with starting PrEP?

____________________________

Thank you!
