# Supplementary material for: Online Navigation for Pre-Exposure Prophylaxis via PleasePrEPMe Chat for HIV Prevention: Protocol for a Development and Use Study
Source: JMIR Res Protoc. 2020 Sep 22;9(9):e20187. doi: 10.2196/20187 (PMC7539157; doi:10.2196/20187)
Supplement: Multimedia Appendix 3 [file resprot_v9i9e20187_app3.docx]

|  | 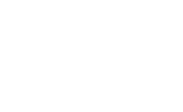 |
| --- | --- |
| **Survey 4:**  **Retrospective Survey** |  |

**Thank you for your time! Your answers are confidential.**

*** Required**

**Section 1**

1. Email address *

____________________________

1. Do you remember chatting with PleasePrEPMe?*

*Mark only one:*

( ) Yes – *go to section 2*

( ) No – *go to section 10*

( ) Don’t know – *go to section 10*

**Section 2**

1. PleasePrEPMe helped me:*

*Select all that apply*

Better understand PrEP or PEP

Find a provider

Find info on paying for PrEP

Find other informational resources and/or links

Understand the California PrEP Assistance Program (CA PrEP-AP)

PleasePrEPMe was not useful

Other: ____________________________

1. Did you contact PleasePrEPMe for yourself or for a client?*

*Mark only one:*

( ) Yourself – *go to section 3*

( ) A client – *go to section 7*

**Section 3**

**If you contacted PleasePrEPMe for yourself**

1. After you contacted PleasePrEPMe, did you start PrEP or PEP?*

*Mark only one:*

( ) Yes – *go to section 4*

( ) No – *go to section 5*

( ) Don’t know – *go to section 5*

**Section 4**

**You started PrEP or PEP**

1. Was PleasePrEPMe able to help you with getting or staying on PrEP or PEP?*

*Mark only one:*

( ) Yes

( ) No

1. If you started POST-exposure prophylaxis (PEP) after contacting PleasePrEPMe, how satisfied were you with your decision?*

Very satisfied

Satisfied

Neither satisfied or dissatisfied

Dissatisfied

Very dissatisfied

Not applicable

1. If you started PRE-exposure prophylaxis (PrEP) after contacting PleasePrEPMe, how satisfied were you with your decision?*

Very satisfied

Satisfied

Neither satisfied or dissatisfied

Dissatisfied

Very dissatisfied

Not applicable

1. If you started PrEP after your contact with PleasePrEPMe and have since stopped, what was the reason(s)?*

____________________________

**Section 5**

**You did not start PrEP or PEP**

1. What was the reason(s) for not starting PrEP or PEP after your contact with PleasePrEPMe?*

____________________________

**Section 6**

**How can PleasePrEPMe help?**

1. How is your life better after contacting PleasePrEPMe?*

____________________________

1. How else might PleasePrEPMe be helpful?*

____________________________

**Section 7**

**If you contacted PleasePrEPMe for a client**

1. After you contacted PleasePrEPMe, did your client make decisions about PrEP or PEP?*

*Mark only one:*

( ) Yes – *go to next question*

( ) No – *go to section 8*

( ) Don’t know – *go to section 8*

1. Was PleasePrEPMe able to help you help your client make decisions about PrEP or PEP?*

( ) Yes

( ) No

( ) Don’t know

**Section 8**

**How can PleasePrEPMe help?**

1. Please rate your satisfaction with PleasePrEPMe’s knowledge level:*

Very satisfied

Satisfied

Neither satisfied or dissatisfied

Dissatisfied

Very dissatisfied

Not applicable

1. Please rate your satisfaction with PleasePrEPMe’s referrals to resources:*

Very satisfied

Satisfied

Neither satisfied or dissatisfied

Dissatisfied

Very dissatisfied

Not applicable

1. How is your life better after contacting PleasePrEPMe?*

____________________________

1. How else might PleasePrEPMe be helpful to you?*

____________________________

1. How else might PleasePrEPMe be helpful to your clients?*

____________________________

**Section 9**

**Demographics**

1. Your age range is:*

*Mark only one:*

( ) 13-18

( ) 19-24

( ) 25-34

( ) 35-44

( ) 45-54

( ) 55+

1. You identify as:*

*Select all that apply*

American Indian or Alaska Native

Asian

Black or African American

Hispanic, Latino, or Spanish origin

Middle Eastern or North African

Native Hawaiian or Other Pacific Islander

White

Multiple races

Other: _______________________

1. You identify your sexual orientation as:*

*Select all that apply*

Bisexual

Gay/Lesbian/Same-Gender Loving/Queer

Not sure

Straight/Heterosexual

Decline to answer

1. Your current gender identity is:*

*Select all that apply*

Female

Genderqueer/Gender Non-binary

Male

Trans Female

Trans Male

Decline to answer

1. Your sex assigned at birth was:*

*Mark only one:*

( ) Male

( ) Female

( ) Decline to answer

1. Would you like to be entered into a drawing for a $100 Visa gift card?

*Mark only one:*

( ) Yes

( ) No

**Section 10**

**Let’s keep in touch**

1. May we contact you in the future? (We don’t have current plans to follow up, but would like to have your permission!)*

*Mark only one:*

( ) Yes, it is ok to contact me

( ) No, please do not contact me again
