# Supplementary material for: Online Navigation for Pre-Exposure Prophylaxis via PleasePrEPMe Chat for HIV Prevention: Protocol for a Development and Use Study
Source: JMIR Res Protoc. 2020 Sep 22;9(9):e20187. doi: 10.2196/20187 (PMC7539157; doi:10.2196/20187)
Supplement: Multimedia Appendix 4 [file resprot_v9i9e20187_app4.docx]

|  |  |
| --- | --- |
| 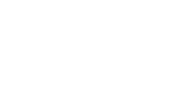**Survey 3:**  **Internal Quality Assurance Review Survey** |  |

1. Name of navigator on chat

____________________________

1. Date of chat

____________________________

1. Transcript number

____________________________

1. Did the navigator understand what the consumer wanted/needed?

*Mark only one:*

( ) Yes

( ) No

1. Were the consumer's needs addressed?

*Mark only one:*

( ) Yes

( ) No

( ) There were missed opportunities

1. If there were missed opportunities, explain:

____________________________

1. Was the Contact Log Entry Form completed accurately?

*Mark only one:*

( ) Yes

( ) No

1. If no, please explain what needs improvement:

____________________________

1. Was the consumer's contact information collected?

*Mark only one:*

( ) Yes

( ) No

1. Was the immediate follow-up survey sent?

*Mark only one:*

( ) Yes

( ) No

1. Was the immediate follow-up survey completed?

*Mark only one:*

( ) Yes

( ) No

1. Was the 14-day follow-up survey sent? (leave blank and fill out in Contact Log Entry Form response sheet 14 days after Contact Log completed)

*Mark only one:*

( ) Yes

( ) No

1. Was the 14-day follow-up survey completed? (leave blank and fill out 14 days after Contact log completed)

*Mark only one:*

( ) Yes

( ) No

1. Was there anything positive from this Contact to highlight?

____________________________

1. What suggestions would you make for improvement (include here: briefer responses, more timely responses, use of transitional wording, more inquisitiveness, clarity etc.)?

____________________________

1. List the suggested action items associated with this QA log and responsible parties. ____________________________
